# Neural network reconstruction using kinetic Ising models with memory

**DOI:** 10.1186/1471-2202-12-S1-P274

**Published:** 2011-07-18

**Authors:** Aree Witoelar, Yasser Roudi

**Affiliations:** 1 Kavli Institute for Systems Neuroscience and Centre for the Biology of Memory, NTNU, Trondheim, Norway; 2 Nordic Institute for Theoretical Physics, Stockholm, Sweden

## 

Ising models with simple Markov chain kinetics have been recently introduced as a tool for inferring asymmetric interactions in neuronal networks [1]. In such an approach, one discretizes time and uses the spike pattern at time step *t* to predict the pattern at time step *t*+*1* and infer the effective interaction between neurons *J*(*i*,*j*) which influences these dynamics. It is however a priori hard to justify that the effect of spikes in only one time bin from the temporal discretization determines the future state of the system. What happens if we use shorter/longer time bins than the characteristic time steps of the network? How do the inferred couplings change if we allow for interactions with memory of multiple past time steps? To answer these questions, we extend the kinetic Ising approach to higher-order Markov chains by introducing time-delayed interactions using (1) a set of couplings derived from scaling the original *J*(*i*,*j*) and (2) an auxiliary set of couplings *K*(*i*,*j*). A model of this sort is closely related to the Generalized Linear Model and its simplicity allows for detailed analysis of the model parameters.

**Figure 1:**
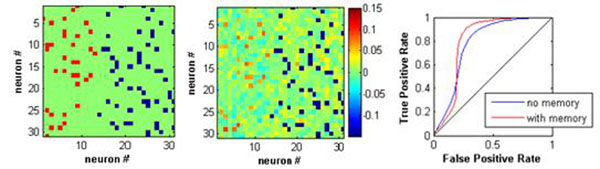
Left: Original network with excitatory (red) and inhibitory connections (blue). Middle: Inferred connectivity using kinetic Ising models with memory. Colors mark their connection strengths. Right: Radio operating characteristics (ROC) curves of inferred excitatory connections.

We apply this extended kinetic Ising model to two types of data sets: (1) a realizable case of randomly-connected Ising network with memory of past states and (2) a realistic cortical network simulation with cross-correlated Hodgkin-Huxley dynamics [2]. In the first case, we test the accuracy from assuming simple Markov chain kinetics to reconstruct higher-order networks using an exact iterative algorithm and mean-field approximations, such as in [1]. In the second case, we aim to identify true synaptic connections in a sparse network. The results show that, not surprisingly, the quality of inferring connections sharply decays at short and long time bins. The effect of adding memory is different depending on the time bin size and there is an optimal combination of time bin and memory, indicating strong interactions at specific time delays. The left panel in Figure 1 illustrates connections between 30 neurons out of the original network composed of 500 excitatory neurons and 500 inhibitory neurons. The inferred network using memory is shown in the middle panel. To have stable balanced states, the excitatory connections are somewhat weaker than the inhibitory ones and are harder to detect using a one-step Markov chain. The time-delayed interactions *K*(*i*,*j*) particularly improve the identification of these weak excitatory connections; see the right panel of Figure 1. Furthermore, *K*(*i*,*j*) is in general linearly dependent on *J*(*i*,*j*) when this connection in the original network is excitatory, but they are less correlated when the connection is inhibitory. This demonstrates that the network has multiple characteristic dynamics which cannot be explained by simple kinetic Ising models.

The authors would like to thank John Hertz for providing the key simulations for the cortical column [2].
